# Criteria for Viability Assessment of Discarded Human Donor Livers during *Ex Vivo* Normothermic Machine Perfusion

**DOI:** 10.1371/journal.pone.0110642

**Published:** 2014-11-04

**Authors:** Michael E. Sutton, Sanna op den Dries, Negin Karimian, Pepijn D. Weeder, Marieke T. de Boer, Janneke Wiersema-Buist, Annette S. H. Gouw, Henri G. D. Leuvenink, Ton Lisman, Robert J. Porte

**Affiliations:** 1 Section of Hepatobiliary Surgery and Liver Transplantation, Department of Surgery, University of Groningen, University Medical Center Groningen, Groningen, The Netherlands; 2 Surgical Research Laboratory, University of Groningen, University Medical Center Groningen, Groningen, The Netherlands; 3 Department of Pathology, University of Groningen, University Medical Center Groningen, Groningen, The Netherlands; Centre Hospitalier Universitaire Vaudois (CHUV), Université de Lausanne, Switzerland

## Abstract

Although normothermic machine perfusion of donor livers may allow assessment of graft viability prior to transplantation, there are currently no data on what would be a good parameter of graft viability. To determine whether bile production is a suitable biomarker that can be used to discriminate viable from non-viable livers we have studied functional performance as well as biochemical and histological evidence of hepatobiliary injury during *ex vivo* normothermic machine perfusion of human donor livers. After a median duration of cold storage of 6.5 h, twelve extended criteria human donor livers that were declined for transplantation were *ex vivo* perfused for 6 h at 37°C with an oxygenated solution based on red blood cells and plasma, using pressure controlled pulsatile perfusion of the hepatic artery and continuous portal perfusion. During perfusion, two patterns of bile flow were identified: (1) steadily increasing bile production, resulting in a cumulative output of ≥30 g after 6 h (high bile output group), and (2) a cumulative bile production <20 g in 6 h (low bile output group). Concentrations of transaminases and potassium in the perfusion fluid were significantly higher in the low bile output group, compared to the high bile output group. Biliary concentrations of bilirubin and bicarbonate were respectively 4 times and 2 times higher in the high bile output group. Livers in the low bile output group displayed more signs of hepatic necrosis and venous congestion, compared to the high bile output group. In conclusion, bile production could be an easily assessable biomarker of hepatic viability during *ex vivo* machine perfusion of human donor livers. It could potentially be used to identify extended criteria livers that are suitable for transplantation. These *ex vivo* findings need to be confirmed in a transplant experiment or a clinical trial.

## Introduction

Donor liver shortage remains a limiting factor in liver transplant programs in most parts of the world. In an attempt to reduce the discrepancy between donor liver availability and demand, criteria for organ acceptance have gradually widened with increasing acceptance of livers that carry a higher risk of early graft failure or transmission of an infectious or malignant disease (so called extended criteria donor (ECD) livers). The types of ECD livers most frequently considered for transplantation are livers with mild-moderate steatosis, livers from older donors or donors with a high body mass index, and livers donated after cardiac death (DCD) [1], [2], [3]. Although livers from ECD donors are increasingly considered for transplantation, many of them are still declined. A recent study in the US has shown that the proportion of donor livers not used for transplantation is increasing since 2004 [4]. The proportion of nonuse attributable to DCD increased from 9% in 2004 to 28% in 2010, probably because in many cases the risk of early graft failure after transplantation is considered to be too high [4].

The decisions to either accept or decline a potential donor liver for transplantation is currently based on the interpretation of donor data, obtained before or during procurement, by the physician. Parameters such as donor past medical history, last known laboratory values, findings during liver procurement, and other procurement variables such as expected ischemia times primarily determine acceptability of the graft. Once a donor liver is retrieved and stored in an organ box for transportation functional assessment is no longer possible until after transplantation. The uncertainty about how much additional damage a liver will sustain during the hours of cold storage poses an important hurdle for accepting many ECD livers.

During the past decade, machine perfusion of donor livers has received increasing attention as a tool to improve organ preservation and improve outcome after transplantation [5], [6], [7], [8], [9]. Several experimental studies have shown superiority of machine perfusion compared to static cold storage with respect to reduction of ischemia/reperfusion (IR) injury [10], [11], [12]. Apart from providing better graft protection against IR injury, machine perfusion provides the possibility of functional assessment of a liver graft short before implantation in a recipient. Although machine perfusion can be performed at various temperatures, only normothermic oxygenated machine perfusion (NMP) may allow a full functional assessment of an organ prior to transplantation. During NMP the liver is offered physiological amounts of oxygen and nutrients supporting a full functional metabolic activity [13]. The possibility of functional assessment of an ECD liver after static cold storage and transportation would be of great importance in the judgment of livers that would otherwise be declined for transplantation based on the current criteria.

Despite the growing amount of literature on the role of machine perfusion as an alternative and better preservation method compared to static cold storage, there are no data on what would be reliable parameters for functional assessment of human donor livers during NMP. Based on a porcine model of normothermic liver perfusion, Imber *et al.* have suggested that bile production is directly attributed to liver viability and could therefore be used as a predictive marker of liver function [11]. In addition, bile production has long been recognized as an important clinical parameter to predict early graft dysfunction (including primary non-function and delayed graft function) after liver transplantation [14]. We, therefore, hypothesized that bile production during NMP of human donor livers is a suitable and easy to assess biomarker of hepatic viability that can be used to discriminate a potentially transplantable from a non-transplantable graft. To test this hypothesis we have studied functional performance as well as biochemical and histological signs of hepatobiliary injury during *ex vivo* NMP of human donor livers that were declined for transplantation. Secondary aim of this study was to determine the minimal duration of NMP needed to discriminate viable and potentially transplantable livers from non-viable livers.

## Materials and Methods

### Liver Donors

Between May 2012 and May 2013 twelve human livers that were declined for transplantation by all three liver transplant centers in The Netherlands, as well as other centers within the Eurotransplant region, were included in this study. Of these, ten were obtained from a DCD donor and two were obtained from donors after brain death (DBD). The donor risk index (DRI) was used to assess the chance of graft failure within three months after transplantation [15]. Livers were retrieved using a standard surgical technique of *in situ* cooling and flush-out with ice cold preservation fluid (University of Wisconsin [UW] or histidine–tryptophan–ketoglutarate [HTK] solution). The surgical procedure was not started until after a five minute ‘no touch’ period following declaration of cardiac arrest and circulatory death in case of a DCD donor. In case of DBD liver procurement the administration of 25.000 units of heparin was given intravenously before cross clamping. The same dose of heparin was added to the preservation solution in case of DCD liver procurement. Livers were subsequently packed and stored on ice and transported to our center. In all cases, informed consent to use a donor liver for this study for this study was provided from the relatives. The study protocol was approved by the medical ethical committee of the University Medical Center Groningen and the *Nederlandse Transplantatie Stichting*, the competent authority for organ donation in the Netherlands.

### Normothermic Oxygenated Machine Perfusion

Upon arrival at our center, cold preserved livers were prepared on the back table for normothermic oxygenated machine perfusion as described previously [13]. NMP was initiated using a CE marked (European Union certification of safety, health and environmental requirements) device that enables dual perfusion via both the hepatic artery and the portal vein in a closed circuit (Liver Assist Organ Assist, Groningen, Netherlands). Livers were perfused for 6 h with a perfusion solution based on heparinized human plasma and red blood cells fortified with nutrients, trace elements and antibiotics as described previously [13]. Two rotary pumps provided pulsatile flow to the hepatic artery and a continuous flow to the portal vein. Two hollow fiber membrane oxygenators provided oxygenation of the perfusion solution, as well as removal of CO_2_. The system was temperature and pressure controlled, allowing auto-regulation of the blood flow through the liver. Pressure was limited to a mean of 60 mmHg in the hepatic artery and 11 mmHg in the portal vein. The temperature was set to 37°C and a new sterile disposable set of tubing, reservoir and oxygenators was used for each liver. Before connecting the liver to the device, the perfusion fluid was primed with the addition of an 8.4% sodium bicarbonate solution to obtain a stable physiological pH. A summary of the composition of perfusion fluid prior to initiation of NMP is provided in Table 1.

**Table 1:** Biochemical Composition of Perfusion Fluid Used For Normothermic Machine Perfusion of Donor Livers.

*Variable*	*Median*	*IQR*	*Reference values in blood*
pH	7.40	7.34–7.45	7.35–7.45
pCO_2_ (kPa)	4.1	3.5–4.6	4.6–6.0
pO_2_ (kPa)	71	65–75	9.5–13.5
sO_2_ (%)	100	99–100	96–99
HCO3- (mmol/L)	19	17–21	21–25
Base Excess (mmol/L)	−4.6	−6.7–−3.5	−3 to 3.0
Na^+^ (mmol/L)	150	145–154	135–145
K^+^ (mmol/L)	4.4	3.8–5.6	3.5–5.0
Free Ca^2+^ (mmol/L)	0.67	0.61–0.72	1.15–1.29
Glucose (mmol/L)*	14	13–15	4–9
Lactate (mmol/L)*	6	6–7	0.5–2.2
Hemoglobin (mmol/L)*	4.7	4.6–4.9	8.7–10.6
Albumin (mmol/L)	31	29–33	35–50
Chloride (mmol/L)	97	91–98	97–107
Urea (mmol/L)	3.5	2.9–3.6	2.5–7.5
Phosphate (mmol/L)	1.8	1.5–2.2	0.7–1.5
Magnesium (mmol/L)	0.55	0.51–0.63	0.70–1.00
Alanine-aminotransferase (U/L)	9	8–11	0–45
Aspartate-aminotransferase (U/L)	13	13–17	0–40
Alkaline phosphatase (U/L)	24	23–28	0–120
Gamma-glutamyltransferase (U/L)	9	7–16	0–40
Lactate dehydrogenase (U/L)	101	93–114	0–250
Total bilirubin (µmol/L)*	2	2–3	0–17

* To convert values for glucose to mg/dL, multiply by 18.02. To convert values for lactate to mg/dL, multiply by 9.01. To convert values for hemoglobin to g/dL, multiply by 1.650. To convert the value for bilirubin to mg/dL, divide by 17.1.

### Assessment of Hepatobiliary Function and Injury

Bile samples were collected from a catheter in the donor common bile duct and bile production was measured gravimetrically at 30 min intervals. Bile production was expressed as g/30 min. Concentration of bilirubin in bile was determined as a marker of hepatic secretory function, using standard biochemical methods. Biliary concentration of bicarbonate and glucose were determined as markers of biliary epithelial cell (cholangiocyte) function. For this purpose, bile samples were collected under mineral oil and analyzed immediately using an ABL800 FLEX analyzer (Radiometer, Brønhøj, Denmark).

During NMP, samples were taken from the perfusion fluid at 30 min intervals and analyzed immediately for blood gas parameters (pO_2_, pCO_2_, sO_2_, HCO^3−^ and pH) and for biochemical parameters (glucose, calcium, lactate, potassium, sodium, and hemoglobin) by an ABL800 FLEX analyzer (Radiometer, Brønhøj, Denmark). Oxygen consumption was calculated based on the difference between the venous oxygen content and the arterial oxygen content of the perfusion fluid. The oxygen content of the perfusion fluid was calculated by adding the free dissolved oxygen fraction to the Hb-bound oxygen fraction using the following formula: O_2_ cont  =  (pO_2_ × K) + (sO_2_ × Hb × c), where pO_2_ is partial oxygen pressure in kPa, K equals 0,027 for O_2_ in water at 37°C, sO_2_ is the saturation expressed as a fraction, Hb is the concentration in mmol/L and c equals 91,12 mlO_2_/mmol for the oxygen binding capacity of hemoglobin. Oxygen consumption was expressed in mlO_2_/min/kilogram liver tissue. Next to this, hepatic concentration of adenosine-5′-triphosphate (ATP) was used as an indicator of the energy status of the liver grafts. Liver samples were immediately frozen in liquid nitrogen. Frozen tissue was cut into 20 µm slices and a total amount of ±50 mg was homogenized in 1 mL of SONOP (0.372 g EDTA in 130 mL H_2_O and NaOH (ph 10.9)+370 mL 96% ethanol) and sonoficated. Precipitate was removed by centrifugation (13,000 rcf for 10 min). Supernatant was diluted with SONOP to attain a protein concentration of 200–300 µg/mL (Pierce BCA Protein Assay Kit, Thermo Scientific, Rockford, IL) and mixed with 450 µL of 100 mM phosphate buffer (Merck; ph 7.6–8.0). Fifty microliters of phosphate buffered supernatant was used for ATP measurement using ATP Biolominescence assay kit CLS II (Boehringer, Mannheim, Germany) and a luminometer (Victor^3^ 1420 multilabel counter, PerkinElmer). ATP concentrations were calculated from a calibration curve constructed on the same plate, corrected for amount of protein, and values were expressed as µmol/g protein.

In addition, plasma from the perfusion fluid was collected (after 5 min centrifugation at 2700 rpm at 4°C), frozen and stored at −80°C for determination of alkaline phosphatase (ALP), gamma-glutamyl transferase (gamma-GT), alanine aminotransferase (ALT), lactate dehydrogenase (LDH), total bilirubin, and albumin, using standard biochemical methods.

### Histological Evaluation

Biopsies were obtained from the liver grafts before and after 6 h of machine perfusion and stored in formalin for histological evaluation. Paraffin-embedded slides of liver biopsies were prepared for hematoxylin and eosin (H&E) staining, and assessed in a semi-quantitative fashion for the presence or absence of venous congestion or >30% hepatocellular necrosis. All liver and slides were examined in a blinded fashion by an experienced liver pathologist (ASHG) using light microscopy.

### Statistical Analysis

Continuous variables are presented as medians and interquartile range (IQR). Categorical variables are presented as number and percentage. Continuous variables were compared between groups using the Mann-Whitney U test. Categorical variables were compared with the Pearson chi-square. Total course of ATP concentration starting at baseline (before NMP) through 6 h of NMP was analyzed between the groups by comparing the area under the curve (AUC, using the trapezium rule). A p-value <0.05 was considered to indicate statistical significance. All statistical analyses were performed using SPSS software version 16.0 for Windows (SPSS, Inc., Chicago, IL).

## Results

### Bile Production as Discriminating Variable during Machine Perfusion

**Figure 1:**
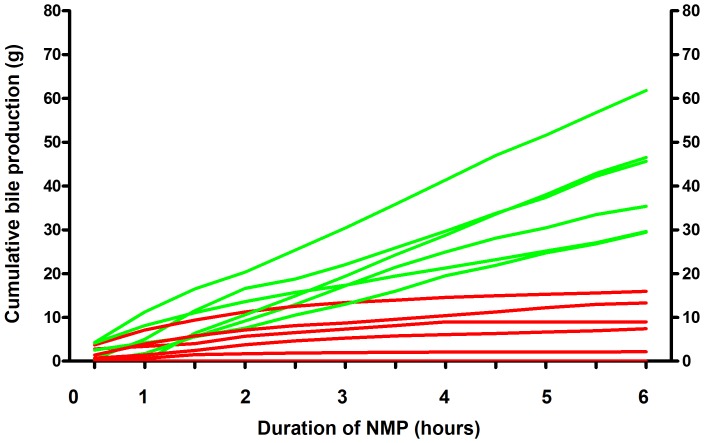
Cumulative bile production during *ex vivo* normothermic machine perfusion of human donor livers. Presented are individual values for 12 livers that were declined for transplantation. *Ex vivo* machine perfusion and viability testing was started after a median cold storage of 6.5 hours. Two distinct patterns of bile flow could be identified: 1) a steadily increasing bile production, resulting in a cumulative bile output of ≥30 g during the 6 h of perfusion (green lines), and 2) an initially increasing bile production during the first 2–3 hours, followed by a diminishing production, resulting in a cumulative bile production in 6 h <20 g (red lines).

First aim of this study was to determine whether bile production is a suitable marker of hepatic viability that can be used during NMP to discriminate a potentially transplantable from a non-transplantable graft. Therefore, we determined the evolution of bile production during 6 h of NMP for all twelve livers. Two distinct patterns of bile flow could be identified: 1) a steadily increasing bile production, resulting in a cumulative bile output of ≥30 g during the 6 h of perfusion, and 2) an initially increasing bile production during the first 2–3 hours, followed by a diminishing production, resulting in a cumulative bile production in 6 h <20 g (Figure 1). Based on this finding of two distinct profiles of bile production a cutoff value of 20 g cumulative bile production during 6 h of NMP was chosen to separate high from low bile output. There were six livers in each group and these two groups were used for further analyses.

A comparison of donor characteristics between livers with a high bile output versus livers with a low bile output during *ex vivo* machine perfusion is provided in Table 2. There were no statistically significant differences for any of these variables. Most livers were declined for transplantation because of a combination of DCD and age (>60 years) or DCD and high BMI. Two livers were declined because of macrovesicular steatosis >30% and both livers were in the low bile output group. It was obvious from this comparison that one would not have been able to identify livers with a high versus low bile output before organ procurement based on conventional donor characteristics alone.

**Table 2:** Donor Characteristics.

	Total group	Low Bile Output	High Bile Output	P-value
	(n = 12)	(n = 6)	(n = 6)	
**Type of donor**				0.12
DCD	10 (83%)	4 (67%)	6 (100%)	
DBD	2 (17%)	2 (33%)	0 (0%)	
**Age (years)**	61 (50–64)	55 (48–65)	63 (51–65)	0.47
**Gender**				0.22
Male	8 (67%)	3 (50%)	1 (17%)	
Female	4 (33%)	3 (50%)	5 (83%)	
**Height (m)**	1.77 (1.67–1.80)	1.77 (1.64–1.81)	1.78 (1.71–1.81)	0.69
**Weight (kg)**	88 (76–98)	90 (85–100)	78 (75–95)	0.20
**Reason for rejection**				0.25
DCD + age>60 years	5 (41%)	1 (17%)	4 (67%)	
DCD + high BMI	3 (25%)	2 (33%)	1 (17%)	
DCD + other reason*	2 (17%)	1 (17%)	1 (17%)	
Severe steatosis**	2 (17%)	2 (33%)	0 (0%)	
**Donor Risk Index**	2.35 (2.01–2.54)	2.48 (2.23–2.61)	2.20 (1.83–2.42)	0.35
**ALT (U/L)** ***	38 (24–59)	59 (34–104)	25 (14–49)	0.05
**GGT (U/L)** ***	90 (39–130)	111 (62–144)	65 (30–130)	0.27
**Prothrombin time (sec)**	14.0 (12.3–16.3)	15.0 (14.0–16.8)	12.9 (9.1–39.7)	0.20
**Preservation Solution**				1.00
UW solution	6 (50%)	3 (50%)	3 (50%)	
HTK solution	6 (50%)	3 (50%)	3 (50%)	
**Time between switch-off and cardiac death (min)**	24 (15–52)	30 (2–53)	24 (17–53)	0.27
**Donor warm ischemia time (min)** †	17 (16–20)	19 (9–26)	17 (15–18)	0.31
**Total donor warm ischemia (min)** †	43 (33–71)	51 (27–72)	43 (34–68)	0.35
**Cold ischemia time (min)**	389 (458–585)	530 (431–750)	409 (363–473)	0.11
**Liver weight (kg)**	2.09 (1.60–2.24)	2.17 (1.60–2.31)	2.03 (1.71–2.18)	0.63

* One DCD donor with history of iv drug abuse (low bile output group) and one donor with prolonged s0_2_<30% after withdrawal of life support (high bile output group).

** defined as macrovesicular steatosis with more than 60% of hepatocytes involved.

*** last known value before procurement.

† Donor warm ischemia times was defined as the time interval between cardiac arrest and start of *in situ* cold perfusion. Total donor warm ischemia time was defined as the time interval between switch -off and start of *in situ* cold perfusion.

Continuous variables are presented as median and interquartile range, categorical variables are presented as numbers and percentage.

Abbreviations used: DCD, donation after cardiac death; DBD, donation after brain death; ALT, alanine aminotransferase; GGT, gamma glutamate transferase; UW, university of Wisconsin; HTK, Histidine- tryptophan-ketoglutarate.

### Comparison of Hepatic Function and Injury

**Figure 2:**
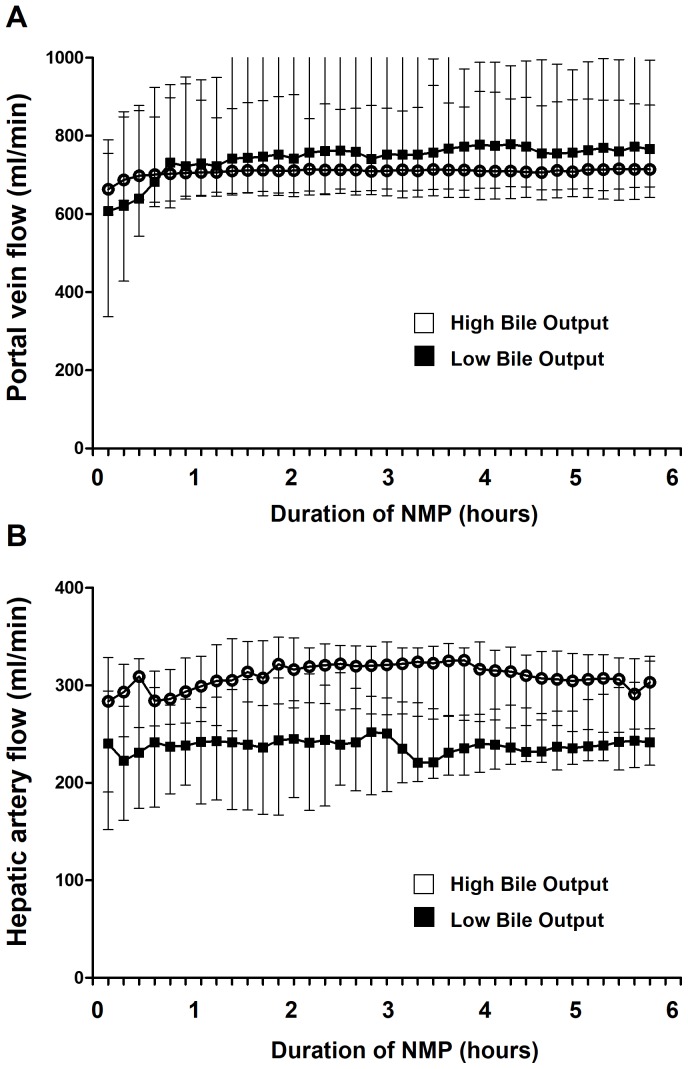
Changes in portal flow (panel A) and arterial flow (panel B) during *ex vivo* normothermic machine perfusion of human donor livers, using a pressure controlled device. Flow in the portal vein and hepatic artery increased rapidly during the first 30 min and flows remained stable thereafter for the entire 6 h perfusion period. There were no significant differences in portal flow and although median arterial flow was constantly lower in livers with a low bile output, compared to the high bile output group, this did not reach statistical significance.

We next examined whether the differences in bile production correlated with other markers of hepatobiliary function and injury during NMP. First, we compared perfusion characteristics between the two groups. During NMP the flow in the portal vein and hepatic artery increased rapidly during the first 30 min and flows remained stable thereafter for the entire 6 h perfusion period (Figure 2). There were no significant differences in portal flow and although median arterial flow was constantly lower in livers with a low bile output, compared to the high bile output group, this did not reach statistical significance.

Biochemical markers of hepatobiliary function and injury after 6 h of *ex vivo* perfusion are presented in Table 3. Most striking differences were significantly higher concentrations of transaminases and a higher potassium level in the perfusion fluid of the low bile output group, compared to the high bile output group. These findings are compatible with a higher degree of IR injury and hepatocellular lysis in the former group. Bicarbonate concentration in perfusion fluid of livers with high bile output was 26 mmol/L (22–28 mmol/L), compared to 18 mmol/L (13–29 mmol/L) in the group of low bile output livers. Although this difference was not statistically different, it should be noted that about 4-times more sodium bicarbonate solution (8.4%) had been added during perfusion in the low bile output group to maintain a physiological pH. After initiation of machine perfusion, glucose and lactate concentrations in the perfusion fluid initially increased in all cases. In the group of livers with high bile output glucose and lactate levels subsequently decreased rapidly and levels were normal at 6 h of NMP. In contrast, glucose and lactate levels in the low bile output group did not normalize during machine perfusion. Albumin levels decreased during all liver perfusions. After 6 h of NMP, albumin levels in the high bile output group were 23 g/L (22–24 g/L), compared to 26 (25–29 g/L) in the low bile output group (Table 3).

**Table 3:** Biochemical Composition of Perfusion Fluid and Bile after 6 hour of *Ex Vivo* Normothermic Machine Perfusion.

	High Bile Output	Low Bile Output	P- value
	(n = 6)	(n = 6)	
***Blood gas variables***			
pH	7.36 (7.25–7.40)	7.34 (7.29–7.40)	1.00
pCO_2_ (kPa)*	6.7 (5.9–7.8)	5.0 (3.4–6.3)	0.08
pO_2_(kPa)*	64 (54–65)	35 (10–67)	0.42
sO_2_ (%)	100 (99–100)	98 (94–99)	**0.04**
HCO_3_^-^ (mmol/L)	26 (22–28)	18 (13–29)	0.20
Added HCO_3_^−^ 8.4% (mL)	8 (0–20)	25 (4–86)	0.24
Base excess (mmol/L)	+0.1 (−3.6–+3.6)	−6.8 (−12.0–−4.0)	0.34
Hemoglobin (mmol/L)*	4.2 (3.7–4.3)	4.3 (4.1-4.6)	0.26
Oxygen consumption** (mlO_2_/min/kilogram liver)	21 (16–22)	60 (27–119)	0.30
***Electrolytes and Metabolites***			
Na^+^ (mmol/L)	154 (143–155)	142 (139–151)	0.26
K^+^ (mmol/L)	4 (2–8)	13 (8–18)	**0.01**
Urea (mmol/L)	14 (11–16)	15 (12–22)	0.63
Albumin (g/L)	23 (22–24)	26 (25–29)	**0.01**
Glucose (mmol/L)*	10 (8–19)	23 (16–32)	0.07
Lactate (mmol/L)*	2 (1–4)	6 (3–11)	**0.03**
***Injury markers***			
ALT (U/L)	2795 (1761–3972)	11074 (6144–16050)	**0.04**
ALP (U/L)	36 (25–44)	154 (82–258)	**0.01**
GGT (U/L)	35 (20–55)	124 (107–187)	0.06
LDH (U/L)	6227 (5151–6703)	22119 (9584–34558)	0.06
Total bilirubin (µmol/L)	3 (3–3)	5 (3–7)	0.20
***Variables measured in bile*** **			
Biliary pH	7.58 (7.56–7.70)	7.37 (7.05–7.71)	0.10
Biliary HCO_3_^−^ (mmol/L)	44 (35–50)	20 (7–41)	0.09
Bilirubin in bile (µmol/L)*	1100 (968–1398)	270 (215–525)	**0.02**

* To convert values for glucose to mg/dL, multiply by 18.02. To convert values for lactate to mg/dL, multiply by 9.01. To convert values for hemoglobin to g/dL, multiply by 1.650. To convert the value for bilirubin to mg/dL, divide by 17.1. To convert kPa to mmHg, multiply by 7.5.

** Peak values during 6 h of machine perfusion.

Abbreviations used: ALT, alanine aminotransferase; ALP, alkaline phosphatase; GGT, gamma-glutamate transferase; LDH, lactate dehydrogenase.

**Figure 3:**
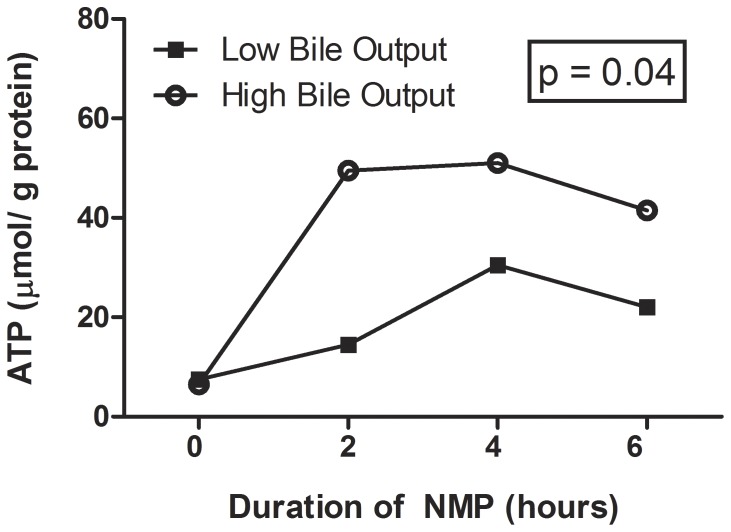
Changes in hepatic energy content as reflected by hepatic ATP content. In contrast to livers with low bile output, livers in the high bile output group showed a significantly higher hepatic ATP content during the course of NMP. (AUC p = 0.04).

At the start of NMP, median pO_2_ in the perfusate was 71 kPa (or 533 mmHg) with an interquartile range of 65–75 kPa (or 488–563 mmHg. After the start of NMP, the pO_2_ dropped in 4 out of the 6 livers with low bile output and median pO_2_ in this group after 6 hours of NMP was 35 kPa (or 263 mmHg). This was not significantly different from pO_2_ values in the high bile output group (Table 3) and this value is still far above the upper limit of normal arterial pO_2_ *in vivo* (13.5 kPa or 101 mmHg). After 6 hr of NMP, there was a small, but significant difference in sO_2_ between the two groups (100% versus 98%), yet median values never fell below the normal range *in vivo* (normal values arterial sO_2_: 96–99%). In parallel with these changes, total hepatic ATP content was significantly higher during the course of NMP in the livers with high bile output, compared to those with low bile output. At baseline, all livers were ATP depleted with a median in the high bile output group 7 µmol/g protein compared to 8 µmol/g in the low bile output group. After 2 h of NMP the ATP had increased to 50 µmol/g in the high bile output group and to 15 µmol/g in the low bile output group. This difference in ATP content persisted during the course of NMP and the AUC analysis revealed statistical significant difference (p = 0.04; Figure 3). In addition, pO_2_ and sO_2_, oxygen consumption was higher in the group of livers with low bile output, compared to those with high bile output, but this did not reach statistical significance.

Biochemical analysis of bile samples during 6 h of NMP revealed a 4-times higher concentration of bilirubin and a 2-times higher biliary concentration of bicarbonate in the high bile output group, compared to the low bile output group (Table 3). These findings indicate that a better secretory function of hepatocytes (bilirubin) coincides with that of cholangiocytes (bicarbonate).

### Histological Comparison

**Figure 4:**
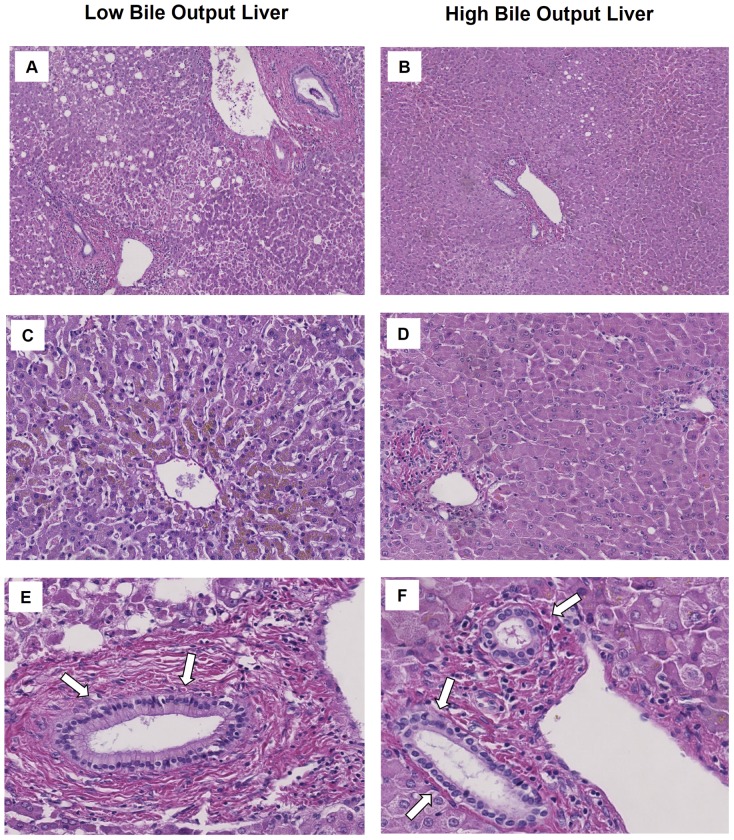
Histology of livers after 6 hours of normothermic machine perfusion. In comparison to livers with high bile output, livers in the low bile output group displayed more signs of hepatic necrosis (panels A and B) and venous congestion (panels C en D). Despite these differences in hepatic parenchymal damage between the two groups, there were no major differences in the degree of biliary damage (panels E and F).

Finally, we compared histology of liver grafts after 6 h of NMP between the two groups. In accordance with the observed differences in biochemical markers of hepatic injury, livers in the low bile output group displayed more signs of hepatic necrosis and venous congestion, compared to the high bile output group (Figure 4 A-D). Venous congestion was present in 5 out of 6 livers (83%) in the low bile output group and in 2 out of 6 livers (33%) in the high bile output group (p = 0.08). Necrosis >30% was observed in 4/6 (66%) of the livers in the low bile output group and in 2/6 (33%) livers in the high bile output group (p = 0.25). Despite these differences in hepatic parenchymal damage between the two groups, there were no major differences in the degree of biliary damage (Figure 4 E-F).

### Minimal Duration of NMP Needed for Viability Assessment

Secondary aim of this study was to determine the minimal duration of NMP needed to discriminate viable and potentially transplantable livers from non-viable livers. For this, we used the individual data on cumulative bile production as depicted in Figure 1. It can be deducted from this figure that livers in the low and high bile output groups can be discriminated from each other as early as 150 min after *ex vivo* machine perfusion. The combination of a cumulative bile production of ≥10 grams at 150 min and a bile production of ≥4 grams in the preceding hour identified 100% of the livers that would be considered as a high bile output liver after 6 h (Table 4). This finding indicates that after cold storage of a donor liver, a short period of 2.5 hours of *ex vivo* assessment during NMP is sufficient to identify a liver that may been preserved well enough to be transplanted successfully.

**Table 4:** Criteria to Assess Bile Production after 2.5 hours of Normothermic Machine Perfusion.

	Liver (Number)	Cumulative Bile Output After 2.5 h (g)	Bile Output Between 1.5 h and 2.5 h (g)	Meets Both Criteria
**Low-bile output**				
	1	6.54	2.52	No
	2	12.53	3.14	No
	3	1.86	0.37	No
	4	4.66	2.19	No
	5	0.00	0.00	No
	6	8.14	2.36	No
**High-bile output**				
	7	18.77	7.22	Yes
	8	12.93	7.33	Yes
	9	14.92	8.50	Yes
	10	10.55	5.00	Yes
	11	15.72	4.66	Yes
	12	25.35	8.85	Yes

Criteria are: 1) Cumulative bile production of ≥10 grams after 2.5 h and 2) a bile production of ≥4 grams in the preceding hour (1.5–2.5 h of perfusion).

## Discussion

Machine perfusion of donor livers is receiving increasing attention as experimental studies have suggested that this method can provide better protection during storage and transportation, compared to static cold storage [10], [11], [12]. Especially ECD livers have been shown to be more susceptible to IR injury, requiring the introduction of novel and more complex preservation techniques [7], [16]. Besides the potential benefits of machine perfusion in providing better protection against preservation injury, this technique also provides the possibility of viability testing of a donor organ prior to transplantation. Pretransplant viability testing may become an important new tool to compensate for the increasing proportion of ECD livers (i.e. livers from donors with advanced age, elevated body mass index, diabetes, or livers from DCD donors), which has resulted in an increasing proportion of non-use of donor livers during the last decade [4].

The main finding in this study is that bile production can be used as an easy assessable marker of liver graft viability during *ex vivo* NMP. Cumulative bile production of ≥30 g during 6 h of NMP was associated with significantly lower release of transaminases and potassium into the perfusion fluid and better hepatobiliary function as reflected by a normalization of glucose and lactate levels and higher biliary secretion of bilirubin. In addition, histology of grafts with a high bile output showed less signs of venous congestion and hepatocellular necrosis, compared to livers with a low cumulative bile output. The second novel finding of this study is that the minimal duration of NMP needed to discriminate viable and potentially transplantable livers from non-viable livers is 2.5 hours. This relatively short time period facilitates a timely selection and preparation of a potential recipient, making this new selection method clinically applicable.

The results of this study open interesting new avenues for the clinical application of *ex vivo* viability testing of ECD livers that, based on conventional criteria, are declined for transplantation. This method has the potential to have a significant impact on the number of donor liver available for transplantation. Of the twelve discarded livers included in this study, 6 (50%) displayed improving function and normalization of hepatobiliary metabolism. Although all livers were declined for transplantation because they were considered ECD livers with a too high risk of primary non-function after transplantation (as indicated by a high DRI), 50% of these may have functioned well after transplantation. All livers were retrieved from a donor outside our hospital and the median duration of cold storage prior to initiation of *ex vivo* viability testing was 6.5 hours. This time sequence can also be expected when this technique is introduced in clinical practice.

In an experimental study using pig livers, Imber *et al* have previously suggested that bile production is probably the most important parameter of liver function [11]. The amount of bile production correlated strongly with the degree of hepatic IR injury. Our experience with discarded human donor livers is in line with this experimental study.

The significant higher release of potassium and ALT in low-bile output livers reflects a higher degree of hepatocellular injury. The absolute concentrations of ALT measured in the perfusion fluid may seem relatively high; however, these results cannot be compared directly with values usually obtained after clinical liver transplantation. First of all, livers were perfused *ex vivo* in a closed circuit and values represent the cumulative release of ALT without any clearance from the system. Secondly, the perfusion circuit contained only 2 liters compared to an average of 5 liters of blood *in vivo*.

In addition to bile production alone, good liver function was reflected by a normalization of glucose and lactate levels in the perfusion fluid, as well as an increasing production of bicarbonate in the livers with high bile output. The latter was reflected by an increasing median concentration of bicarbonate in perfusion fluid from 19 mmol/L at baseline to 26 mmol./L after 6 h of NMP in the high bile output group. In the low bile output group median bicarbonate concentration in the perfusion fluid at 6 h was only 18 mmol/L, despite the addition of a 4-times higher amount of sodium bicarbonate 8.4% during perfusion to maintain a physiologic pH. These finding are in accordance with a previous animal study that has indicated that autoregulation of the acid-base balance is a reflection of a well-functioning liver [17].

During 6 h of NMP albumin levels decreased in all perfusions. Although levels after 6 h of NMP were slightly (but significantly) lower in the high bile output group compared to the low bile output group, this cannot be used as a marker of hepatic synthetic function because of the overall decline in albumin during all perfusions.

Interestingly, oxygen consumption appeared to be higher in livers with low bile production, compared with those with high bile production. This finding is in agreement with observations made by Imber *et al.* during NMP of porcine livers [11]. These investigators found significantly higher oxygen consumption during NMP of livers that were severely injured after prolonged cold preservation, compared to well preserved donor livers. Imber *et al.* explained this difference in oxygen consumption by the respiratory burst and subsequent oxygen debt that occurs in severely injured post-ischemic livers [11]. In addition, ATP concentration appeared to be significantly higher in livers with high bile production reflecting a higher energy status upon reperfusion compared to livers with low bile output.

In the current study, we did not add bile salts to the perfusion fluid. Hepatocellular secretion of bile salts into bile canaliculi is an important driving force of bile flow [18], [19]. *In vivo*, bile salts are reabsorbed from the gut and transported back to the liver through the enterohepatic circulation. Bile salts are subsequently secreted again into the bile, causing a choleretic increase in total bile flow. Obviously, this enterohepatic circulation is interrupted during *ex vivo* NMP and this could theoretically lead to bile salt depletion and a subsequent decline in bile production. However, experimental studies using pig livers have shown that bile salt depletion does not occur until after 10 hours of NMP [19]. In the current study, livers were perfused for 6 hours and we did not observe a decline in bile output. Therefore, we do not believe the addition of bile salts is necessary when livers are perfused for less than 10 hours. In fact, hydrophobic bile salts have been demonstrated to play a role in bile duct epithelial injury after liver transplantation and this could be considered an additional argument not to add bile salts to the perfusion fluid [19], [20], [21], [22]. On the other hand, due to the strong choleric effect of bile salts, bile production during *ex vivo* NMP will be higher when bile salts are added to the perfusion fluid. This should be kept in mind when comparing bile output values obtained in different studies.

Bile production is an energy consuming, multi-step process that requires an intact network of sinusoidal cells, hepatocytes and cholangiocytes. Therefore, intuitively, bile production is a strong and reliable indicator of overall liver quality and viability. In clinical liver transplantation, poor initial bile production has been associated with poor outcomes. In one study, graft survival at one year was only 45% for livers that failed to produce bile in the operating room [14]. In addition to bile volume, we have shown a higher biliary secretion of bilirubin in grafts with high bile output, reflecting a better quality of bile produced by these livers.

Although some studies on kidney and liver machine perfusion have suggested that a decline in arterial flow in a pressure controlled system of machine perfusion can be used as a marker of decreasing graft viability [23], [24], [25], we found stable flows in both high and low bile output livers. Apparently, change in perfusion flow is not a reliable parameter of graft viability in human liver machine perfusion. However, in livers with a low bile production we did observe a lower arterial flow during the entire 6 h of NMP, compared to the low bile output group, but this did not reach statistical significance. In general, we do not advise to use flow values as an indicator of liver damage and viability during human liver machine perfusion.

A limitation of *ex vivo* NMP is the inability to assess bile duct viability. Although the main aim of the current study was to assess hepatocyte viability, ischemic cholangiopathy, resulting in non-anastomotic biliary strictures, remains a major complication after liver transplantation [3]. Unfortunately, reliable markers or other tools that help predict ischemic cholangiopathy before transplantation are still lacking. Therefore, we cannot rule out that some of the livers that we considered viable based on *ex vivo* bile production may still have developed non-anastomotic biliary strictures if they had been transplanted. Clearly, there is a need to develop non-invasive methods that enable assessment of the biliary epithelium during organ preservation and before implantation. An attractive option could be the development of molecular imaging techniques using near-infrared fluorescence that allow a non-invasive assessment of the biliary epithelium. If such molecular imaging techniques are combined with visible light cholangioscopy this could provide a tool for assessment of the biliary tree during NMP [26], [27].

A second limitation of the study is the relative small number of liver grafts. Unfortunately human livers do not come available for research in high numbers. This may have caused a statistical type II error, explaining the trend towards significance for some variables as presented in Table 3.

In conclusion, this study suggests that the assessment of bile production is a discriminative indicator of hepatic function and injury during *ex vivo* NMP of human donor livers. It could potentially be used to identify ECD livers that are declined for transplantation based on donor risk factors, but that may still be suitable for transplantation. These findings need to be confirmed in a clinical trial in which the proposed selection criteria are used to accept ECD livers that would otherwise have been declined for transplantation based on an anticipated poor postoperative function. We are currently preparing such a trial.

## References

[1] Merion RM, Goodrich NP, Feng S (2006) How can we define expanded criteria for liver donors? J Hepatol 45: 484–488. 16905221 10.1016/j.jhep.2006.07.016

[2] McCormack L, Dutkowski P, El-Badry AM, Clavien PA (2011) Liver transplantation using fatty livers: always feasible? J Hepatol 54: 1055–1062. 21145846 10.1016/j.jhep.2010.11.004

[3] Op den Dries S, Sutton ME, Lisman T, Porte RJ (2011) Protection of bile ducts in liver transplantation: looking beyond ischemia. Transplantation 92: 373–379. 21629175 10.1097/TP.0b013e318223a384

[4] Orman ES, Barritt AS 4th, Wheeler SB, Hayashi PH (2013) Declining liver utilization for transplantation in the United States and the impact of donation after cardiac death. Liver Transpl 19: 59–68. 22965893 10.1002/lt.23547 PMC3535500

[5] Bae C, Henry SD, Guarrera JV (2012) Is extracorporeal hypothermic machine perfusion of the liver better than the 'good old icebox'? Curr Opin Organ Transplant 17: 137–142. 22277954 10.1097/MOT.0b013e328351083d

[6] Brockmann J, Reddy S, Coussios C, Pigott D, Guirriero D, et al (2009) Normothermic perfusion: a new paradigm for organ preservation. Ann Surg 250: 1–6. 19561463 10.1097/SLA.0b013e3181a63c10

[7] Dutkowski P, de Rougemont O, Clavien PA (2008) Machine perfusion for 'marginal' liver grafts. Am J Transplant 8: 917–924. 18416733 10.1111/j.1600-6143.2008.02165.x

[8] Hessheimer AJ, Fondevila C, Garcia-Valdecasas JC (2012) Extracorporeal machine liver perfusion: are we warming up? Curr Opin Organ Transplant 17: 143–147. 22273595 10.1097/MOT.0b013e328351082a

[9] Monbaliu D, Brassil J (2010) Machine perfusion of the liver: past, present and future. Curr Opin Organ Transplant 15: 160–166. 20125022 10.1097/MOT.0b013e328337342b

[10] Fondevila C, Hessheimer AJ, Maathuis MH, Munoz J, Taura P, et al (2012) Hypothermic oxygenated machine perfusion in porcine donation after circulatory determination of death liver transplant. Transplantation 94: 22–29. 22691959 10.1097/TP.0b013e31825774d7

[11] Imber CJ, St Peter SD, Lopez de Cenarruzabeitia I, Pigott D, James T, et al (2002) Advantages of normothermic perfusion over cold storage in liver preservation. Transplantation 73: 701–709. 11907414 10.1097/00007890-200203150-00008

[12] Schlegel A, Rougemont O, Graf R, Clavien PA, Dutkowski P (2013) Protective mechanisms of end-ischemic cold machine perfusion in DCD liver grafts. J Hepatol 58: 278–286. 23063573 10.1016/j.jhep.2012.10.004

[13] op den Dries S, Karimian N, Sutton ME, Westerkamp AC, Nijsten MW, et al (2013) Ex vivo normothermic machine perfusion and viability testing of discarded human donor livers. Am J Transplant 13: 1327–1335. 23463950 10.1111/ajt.12187

[14] Markmann JF, Markmann JW, Desai NM, Baquerizo A, Singer J, et al (2003) Operative parameters that predict the outcomes of hepatic transplantation. J Am Coll Surg 196: 566–572. 12691933 10.1016/S1072-7515(02)01907-5

[15] Feng S, Goodrich NP, Bragg-Gresham JL, Dykstra DM, Punch JD, et al (2006) Characteristics associated with liver graft failure: the concept of a donor risk index. Am J Transplant 6: 783–790. 16539636 10.1111/j.1600-6143.2006.01242.x

[16] Pomfret EA, Sung RS, Allan J, Kinkhabwala M, Melancon JK, et al (2008) Solving the organ shortage crisis: the 7th annual American Society of Transplant Surgeons' State-of-the-Art Winter Symposium. Am J Transplant 8: 745–752. 18261169 10.1111/j.1600-6143.2007.02146.x

[17] Reddy SP, Bhattacharjya S, Maniakin N, Greenwood J, Guerreiro D, et al (2004) Preservation of porcine non-heart-beating donor livers by sequential cold storage and warm perfusion. Transplantation 77: 1328–1332. 15167586 10.1097/01.tp.0000119206.63326.56

[18] Portincasa P, Calamita G (2012) Water channel proteins in bile formation and flow in health and disease: when immiscible becomes miscible. Mol Aspects Med 33: 651–664. 22487565 10.1016/j.mam.2012.03.010

[19] Imber CJ, St Peter SD, de Cenarruzabeitia IL, Lemonde H, Rees M, et al (2002) Optimisation of bile production during normothermic preservation of porcine livers. Am J Transplant 2: 593–599. 12201359 10.1034/j.1600-6143.2002.20703.x

[20] Buis CI, Geuken E, Visser DS, Kuipers F, Haagsma EB, et al (2009) Altered bile composition after liver transplantation is associated with the development of nonanastomotic biliary strictures. J Hepatol 50: 69–79. 19012987 10.1016/j.jhep.2008.07.032

[21] Yska MJ, Buis CI, Monbaliu D, Schuurs TA, Gouw AS, et al (2008) The role of bile salt toxicity in the pathogenesis of bile duct injury after non-heart-beating porcine liver transplantation. Transplantation 85: 1625–1631. 18551070 10.1097/TP.0b013e318170f5f7

[22] Hoekstra H, Porte RJ, Tian Y, Jochum W, Stieger B, et al (2006) Bile salt toxicity aggravates cold ischemic injury of bile ducts after liver transplantation in Mdr2+/− mice. Hepatology 43: 1022–1031. 16628673 10.1002/hep.21169

[23] Obara H, Matsuno N, Enosawa S, Shigeta T, Huai-Che H, et al (2012) Pretransplant screening and evaluation of liver graft viability using machine perfusion preservation in porcine transplantation. Transplant Proc 44: 959–961. 22564596 10.1016/j.transproceed.2012.01.104

[24] Nyberg SL, Baskin-Bey ES, Kremers W, Prieto M, Henry ML, et al (2005) Improving the prediction of donor kidney quality: deceased donor score and resistive indices. Transplantation 80: 925–929. 16249740 10.1097/01.tp.0000173798.04043.af

[25] Impedovo SV, Martino P, Palazzo S, Ditonno P, Tedeschi M, et al (2012) Value of the resistive index in patient and graft survival after kidney transplant. Arch Ital Urol Androl 84: 279–282. 23427764

[26] Moon JH, Terheggen G, Choi HJ, Neuhaus H (2013) Peroral cholangioscopy: diagnostic and therapeutic applications. Gastroenterology 144: 276–282. 23127575 10.1053/j.gastro.2012.10.045

[27] Karimian N, op den Dries S, Porte RJ (2013) The origin of biliary strictures after liver transplantation: Is it the amount of epithelial injury or insufficient regeneration that counts? J Hepatol 58: 1065–1067. 23466306 10.1016/j.jhep.2013.02.023

